# Drying Characteristics and Quality Analysis of Medicinal Herbs Dried by an Indirect Solar Dryer

**DOI:** 10.3390/foods11244103

**Published:** 2022-12-19

**Authors:** Anfal Al-Hamdani, Hemanatha Jayasuriya, Pankaj B. Pathare, Zahir Al-Attabi

**Affiliations:** 1 Department of Soils, Water and Agricultural Engineering, College of Agricultural and Marine Sciences, Sultan Qaboos University, Muscat 123, Oman; 2 Department of Food Science and Nutrition, College of Agricultural and Marine Sciences, Sultan Qaboos University, Muscat 123, Oman

**Keywords:** indirect solar dryer, medicinal herbs, drying kinetics, color retention, phenol content

## Abstract

Considering the solar radiation status in Oman, a low-cost, indirect, stand-alone, forced-convective solar dryer was developed to dry medicinal herbs, which are sensitive to direct sun. The hot air flow was obtained using a solar-panel-powered blower and air passing through a black-body solar collector. This drying process could extend the shelf life of herbs while preserving their medicinal and nutritional (physicochemical) properties and adhering to food safety and hygiene practices. This study investigated the benefits of an indirect solar drying technique on the retention of quality attributes of mint and basil used in medicinal applications. Herbs used during drying could be subjected to changes in their physicochemical properties such as color, water activity (Aw), total soluble solids (TSS), phenol content, antioxidant capacity, and moisture content (MC), and, thus, results were compared with fresh herb samples. The dryer chamber-maintained temperature and relative humidity regimes of 30–50 °C and 21–95% and the expected final moisture content (wet basis) was 10%. The dryer showed improved physicochemical quality parameters and the retention of green color with parameter ranges of Aw 0.44–0.63, phenol content (increase) 1705–8994 mg/100 g DM, and antioxidant capacity (increase) 0.61–0.67 µmol/g DM, respectively. This study showed the ability of developed solar dryers to preserve the physicochemical properties of medicinal herbs during drying and can extend to other food products.

## 1. Introduction

Medicinal herbs play an important role in mostly Ayurveda and other eastern medicinal treatments for many current chronic diseases such as Cancer, Crohn’s Disease, Ulcerative Colitis and Cystic Fibrosis. There are many types of medicinal herbs; for example, Basil (*Ocimum basilicum* L.) is an annual plant in the *mint* family (Lamiaceae) that grows throughout the summer. It has been used for millennia and is a vital part of many traditions and rituals [1]. *Basil* is considered a pharmacological herb because it can use as a prophylactic agent (hepatic and gastritis disorders), anticancer activity, Radioprotective activity, antipyretic activity, and antioxidant activity [1]. Mint (*Mentha spicata* L.) is one of the most diverse and widespread dicotyledonous plant groups in the Lamiaceae or *mint* family. *Mint* is used for headaches, skin irritation, diarrhea, and menstrual irritation [1]. Different types of medicinal herbs are grown in Oman, and most of them are sent to markets in fresh form and subjected to a significant number of postharvest losses.

The food industries around the world tend to establish different preservation methods to depreciate pathogenic bacteria, conserve food nutrition value, reduce agricultural waste, and reduce production costs [2]. Open sun drying is considered to be in a direct mode, which means that solar radiation heats the product directly. Open sun drying is the cheapest and simplest method of drying agricultural products, including medicinal herbs. Herbs lose their aroma and become discolored when exposed to direct sunlight, which makes them less appealing to consumers. In addition, pest infestations, bird droppings, rodent attacks, and other occurrences can take place. Mouhoubi et al., [3] stated that agricultural products are very sensitive and perishable at a specific time. The low boiling point compounds present in leaves evaporate during the drying process. It should be noted that oxidation modification affects the nutritional, physical, and chemical contents of dried leaves. A medicinal herb’s quality degrades as a result of the active compounds’ thermal breakdown during the drying [4]. The drying industry has found that solar drying is an effective and reliable method for preserving foods and aromatic plants [5], because it is the indirect mode which uses hot air to heat the product and then the air heats it by solar radiation. Singh et al. [6] found that the ascorbic acid, total chlorophyll content, and color of the dried samples were found to be better in the solar dryer compared with open sun drying. Furthermore, the solar-dried Rupturewort (*Herniaria hirsuta*) samples showed the highest retention of antioxidants when compared with open sun drying [7]

Oman has abandoned solar radiation year-round; being highest in the region [8], Oman’s solar radiation can still be utilized as a renewable energy source for drying agricultural produce minimizing waste and improving shelf life. Furthermore, Oman’s 2040 vision focuses on using eco-friendly renewable energy for possible developmental activities. In addition, the government of Oman is making efforts to promote youth and women’s involvement in small and medium enterprises (SMEs) in Oman. Therefore, drying using low-cost solar energy could be beneficial to the agricultural SMEs. Agricultural products have long been preserved via solar drying. However, as drying is weather-dependent, it can take longer than expected, damaging the product’s quality and causing losses [9,10]. A low-cost, indirect solar dryer could be an alternative to the common method used (i.e., direct sun drying). This technology might be applied locally to boost farmers’ incomes and ensure their access to food and nutrition. Therefore, this research aimed to study the effect of a low-cost, stand-alone, indirect solar dryer prototype in producing high-quality dried medicinal herbs, such as mint and basil. This work also evaluated the drying characteristics and physicochemical properties of dried herbs.

## 2. Materials and Methods

### 2.1. Sample Preparation

The study was carried out on 2 kg leaves of basil (Var. *Sweet Basil*) and 2 kg of mint (Var. *Moroccan Mint*) purchased from the local market in the region of Barka, South Al Batina, Oman. The basil leaves have an initial moisture content of 87.7% and the mint has an initial moisture content of 71%. The leaves were separated from the stems. After sorting, the samples were packed in glass containers and refrigerated (2–4 °C) for a maximum of 12 h.

### 2.2. Drying Process

**Figure 1:**
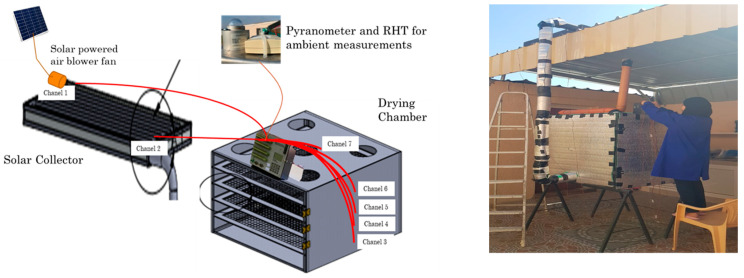
Indirect solar dryer for herbs drying process.

The experiments were conducted in two seasons, the summer season (July, August) and winter season (December, January) in Barka, North Al-Batinah, Oman (23°42′26″ N, 58°09′43.7″ E). In the summer, the solar radiation hours were 11 h, with an average ambiance temperature and relative humidity of 42.5 °C and 44.5%, respectively. In the winter, the solar radiation hours were 8 h, with an average ambiance temperature and relative humidity of 38.3 °C and 30.8%, respectively. Three drying experiments were performed using the developed low-cost indirect solar dryer (four replicates), direct sun, and shade drying, and by using two types of medicinal herbs (basil, and mint). The solar collector of the developed indirect solar dryer uses the black body radiation concept which depends on solar radiation, ambient weather conditions, and airflow settings (Figure 1). Furthermore, the drying chamber follows the thin-layer drying concept. Two other drying methods; direct sun and shade drying were obtained for comparison and weight was obtained periodically using a digital scale with an accuracy of 0.001 g. The drying process was continued until the moisture content of the herb reached 10% (the best value for the dried product, [8]), with knowledge of the initial moisture content. The dried material was protected from light and moisture by being hermetically sealed in plastic vials.

### 2.3. Mathematical Modelling

During experiments, the drying chamber was arranged with thermocouples and humidity sensors with a data logger sampling frequency of 10 min and averaged for each hour synchronizing with the weight measurements. The moisture loss was recorded every 1 h in winter and 0.5 h in the summer seasons. The moisture content against time was used to generate the drying rate curve.

Average moisture content for thin layer drying is presented by the following expression:

(1) $$MR=\frac{M-Me}{Mo-M_{e}}$$

where, *MR* = Moisture ratio

*M_o_* = Initial moisture content, %

*M* = Moisture content, %

The moisture ratio may be simplified to *M*/*M_o_* because of the value of equilibrium moisture content *Me* is very small compared with *M* and *M_o_*.

Among a huge number of drying kinetic models that were used for agricultural products, three models developed for herbs were selected in this study: Lewis, Modified Page-I, and Midilli and Kucuk (Table 1). The selected models were compared with the drying characteristics of medicinal herbs (Basil and Mint). Best-fitting models depended on the highest value of R^2^ and lowest value of Chi-Square (χ^2^) and RMSE.

## 3. Quality Analysis

### 3.1. Measurement of Moisture Content (MC)

The initial moisture content of the fresh herb was determined by Hotbox Oven (Gallenkamp, Cambridge, UK) at 105 °C for 24 h [14]. All drying experiments were conducted; the averages were utilized for further analysis. The MC reduction measurements were taken at regular intervals, and the drying process was continued until the moisture content reached 10%.

### 3.2. Color Measurements

The color quality of the drying herb was evaluated using a portable colorimeter (3nh Precision Colorimeter, NR110, Shenzhen, China) equipped with the D65 illuminant and 10° observer in terms of coordinates L*, a*, and b*. The color brightness coordinate L* is used to determine a color’s whiteness value, and it runs from 0 to 100 (black to white). When negative, the chromaticity coordinate a* determines green, and when positive, it determines red. When positive, the chromaticity coordinate b* determines yellow, and when negative, it determines blue [15]. Color change is expressed as a single numerical number using the total color difference (∆E), hue, and Chroma. In addition, dark green color index (DGCI) values were a more consistent measure of green color than individual RGB values. The color index for dark green was derived as follows [16]:

(2) $$DGCI=\frac{\left[\frac{Hue-60}{60}+\left(1-chroma\right)+\left(1-L^{*}\right)\right]}{3}$$

(3) $$\Delta E^{*}=\sqrt{\Delta L^{*2}+\Delta a^{*2}+\Delta b^{*2}}$$

(4) $$\mathrm{Chroma}=\sqrt{a{*}^{2}+b{*}^{2}}$$

(5) $$\mathrm{Hue}=\tan^{-1}\left(\frac{b*}{a*}\right)$$

### 3.3. Measurement of Water Activity (Aw)

The measurement of the water activity of fresh and dried herbs was done using a water activity meter (Model: 10972, HygroLab C1, Rotronic, Bassersdorf, Switzerland).

### 3.4. Measurement of Total Phenol Content

The measurement of Total Phenol content was measured by the extraction process used for Total Flavonoids Content (TPC) and antioxidant (AO) properties [17]. In the process, 10 g of powdered sample was dissolved in 70 mL of ethanol. After that, the solution was put in ultrasonic for 30 min. The mass of the mixture was taken before and after evaporation using a rotary evaporator. The sticky partial (extracted crude sample) appeared when the evaporation process ended. The microfiltration of 0.45 mm was used to remove any precipitations. The final results were obtained using a standard curve.

The extracted crude sample was diluted with a 100-dilution ratio and then mixed with Folin-Ciocalteu with a ratio of 1:1. After that, 2 mL of 20% NaCO_3_ was added to the previous solution. The mixture was put in boiling water for 5 min. Moreover, Caffeic acid was used as a Standard. Folin was diluted by adding 5 mL of Folin to 5 mL of distilled water and 20% NaCO_3_ was prepared by 20 g of NaCO_3_ to 100 mL of distal water. The final sample was inserted into a UV/VIS spectrometer (Thermo Scientific Fluoroskan Ascent FL, USA) and the absorption was taken at 650 nm.

### 3.5. Measurement of Antioxidant (AO) Content

Total antioxidant capacity (TAC) was measured using the Colorimetric Assay Kit (United States, BioVision, Absorbance (400 or 405 nm) which comprised 0.2 mL Cu^2+^ reagent, 10 mL assay diluent 10 mL protein mask, and 1µmol Trolox standard [18].

Firstly, lyophilized Trolox dissolved in 20 µL of pure Dimethyl sulfoxide (DMSO) and 980 µL of distilled water and mixed properly. After that, 0, 4, 8, 12, 16, and 20 µL of Trolox standard were taken to individual wells. The total volume was adjusted to 100 µL by adding deionized distilled H_2_O to reach 0, 4, 8, 12, 16, and 20 nmol of Trolox standard. Secondly, 0.2 mL Cu^2+^ was mixed with 10 mL assay diluent (1:49), and 5 µL of the extracted sample was added into the well (plate including 96 wells), with 5 µL of the protein mask and 90 µL of Deionized H_2_O. Furthermore, 100 µL of diluted Cu^2+^ reagent was added to all standard and sample wells.

### 3.6. Statistical Analysis

Excel 2016 and R programs for Windows 10 version 21H2 and SPSS 20.0 (International Business Machine Crop., Armonk, NY, USA) were used to conduct all data/statistical analyses. All analyses were carried out using triplicated data gathered during experiments, and results are given as the mean standard deviation (SD). The SPSS 20.0 was used to study the impact of the experimental variables i.e., drying methods (Indirect solar, direct sun, shade drying) and seasons (winter, and summer) on physical (moisture content, and color) and chemical factors (Total soluble solids (*TSS*), water activity (*Aw*), Total Phenol Content, and Antioxidant capacity) by performing mean values which were considered at a 5% significance level (*p* < 0.05). Regression analyses, such as coefficient of determination (*R*^2^) (Equation (6)), chi-square (*X*^2^) (Equation (7)), and root mean square error (*RMSE*) (8), were performed as a primary standard to select the best fit of the tested experimental data. The adequately fitted quality parameters were determined with the highest *R*^2^ and lowest *χ*^2^, and *RMSE*. The following equations were used for parameter estimations [19]:

(6) $$R^{2}=1-\frac{\sum_{i=1}^{n}{\left(MR_{Pre,i}-MR_{exp,i}\right)}^{2}\;}{\sum_{i=1}^{n}{\left(MR_{Pre}-MR_{exp,i}\right)}^{2}\;}$$

(7) $$\chi^{2}=\overset{n}{\underset{i=1}{\sum }}\frac{{\left(MR_{exp,i}-MR_{Pre,i}\right)}^{2}}{N-n}\;$$

(8) $$RMSE=\sqrt{\overset{n}{\underset{i=1}{\sum }}\frac{1}{N}\;{\left(MR_{exp,i}-MR_{Pre,i}\right)}^{2}}$$

## 4. Result and Discussion

### 4.1. Evaluation of Ambient Parameters

The solar radiation in the summer seasons was higher than in the winter, which could enhance the drying characteristic of selected products. On average, the solar collector could increase the dryer chamber temperature by 10 °C. The solar collector’s maximum temperature reached 65.0 °C, and the dryer chamber reached 50.8 °C. There was a 14.26 °C temperature drop between the outlet of the solar collector and the inlet of the drying chamber, as a result of heat lost through the insulated pipes.

### 4.2. Medicinal Herbs’ Moisture Content

**Figure 2:**
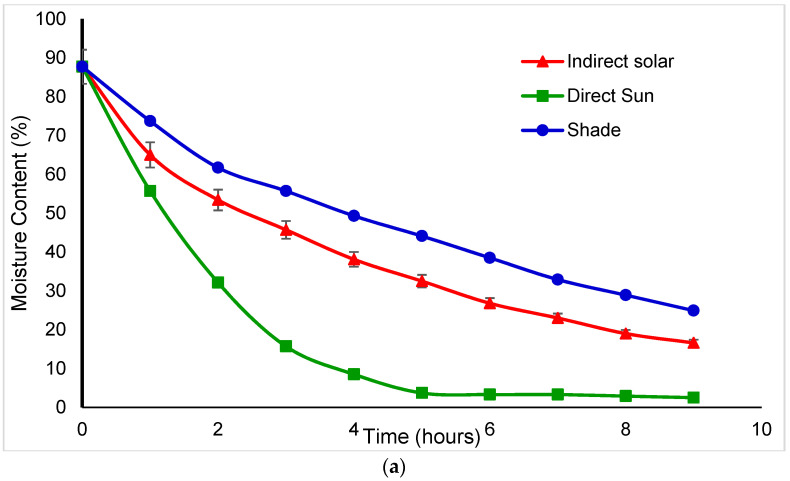

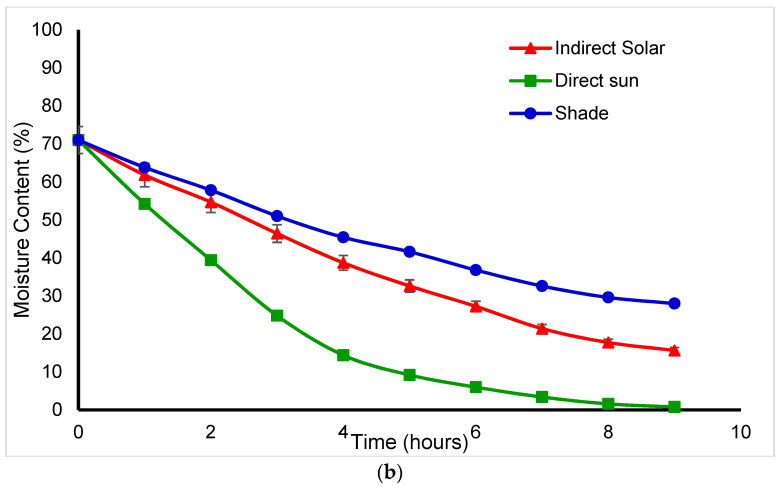
The moisture content reduction in (**a**) Basil and (**b**) Mint during winter.

**Figure 3:**
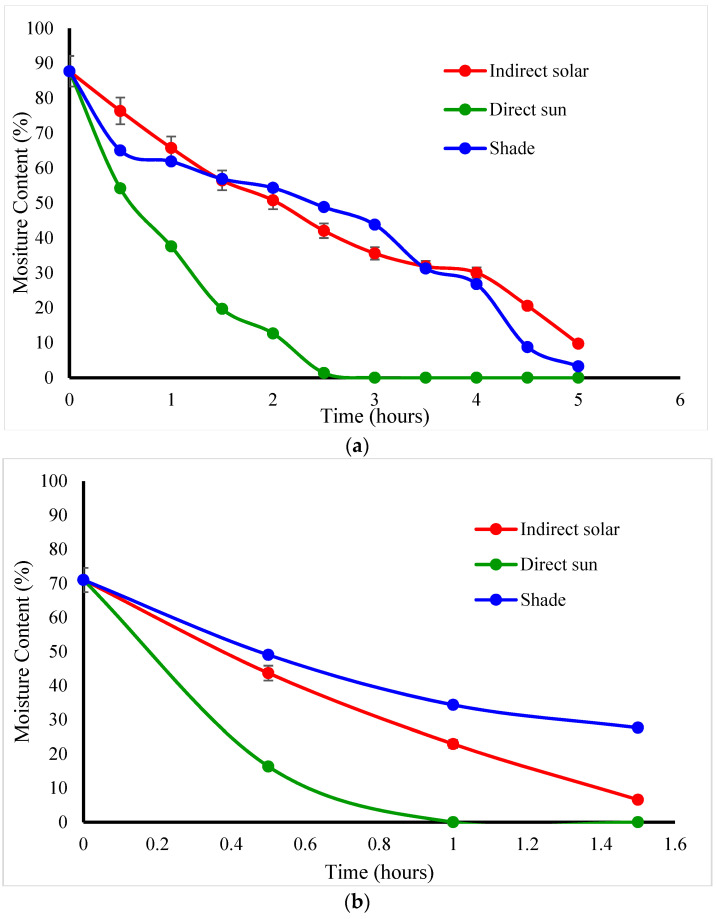
The moisture content reduction in (**a**) Basil and (**b**) Mint in summer.

Fresh basil and mint had wet basis initial moisture contents of 87.7 and 71.0%, respectively. Mint has a higher solid content than basil., It was found that the *basil* mass reduction during the summer season was reduced by 93.8, 77.97, and 84.43%, respectively, for direct sun, indirect, and shade drying, which was indicated by a final moisture content value of 10% for both basil and mint. On the other hand, the winter season recorded 85.52, 71.08, and 62.8% reductions in direct sun, indirect solar dryer, and shade drying, respectively. The reduction in weight during winter was 8%, 7%, and 22% less reduction than during the summer season. The mint mass reduction during the summer season when the temperature reached 55 ^ᵒ^C was reduced by 79.67% and 64.4% for direct sun drying and indirect solar dryer. On the other hand, the winter season showed 70.13% and 55.34% for direct sun and indirect solar dryers, respectively. The reduction in weight during winter was 10% less than during the summer season. However, no difference in weight reduction under shade drying. Direct sun drying caused the most weight loss in *basil*, followed by mint in both seasons. Figure 2 and Figure 3 show the reduction in weight which was due to the loss of solids and water during the drying process in the winter and summer seasons. The statistical study revealed that moisture content was reduced (*p* < 0.05) across all drying processes for medicinal herbs. Kanakidi et al. [20] reported that the best condition to dry the bay leaves using hot air drying at a temperature of 60 °C is to decrease the moisture content to 10% (wb). According to Karami et al. [21] a temperature of 70 °C and air velocity of 2 m/s could significantly shrink the drying time for rosemary. Figure 2a indicates the basil moisture content reduction during winter seasons for three different types of dying methods (open sun, shade, and indirect solar dying). First, using open sun drying, the moisture content of basil was reduced from 87.7% to 10% within 6 h. However, using an indirect solar dyer, the basil takes 14 h to reach 10% of the moisture content. Figure 2b and Figure 3b showed the mint moisture content reduction during winter seasons for three same dying methods (open sun, shade, and indirect solar dying). It is clear that the open sun drying reduces the moisture content of mint from 71% to 10% within 5 h, but the indirect solar dyer takes 12 h. Therefore, the faster dying method was the open sun, indirect solar dryer, and then shade dying. On the other hand, the summer season in Figure 3. showed that the *basil* was dried within 2 h under the open sun and 5 h using indirect solar dyer. The mint drying (Figure 3b) takes 30 min using direct sun and 84 min using a developed dryer.

### 4.3. Drying Kinetic Models of Medicinal Herbs

**Figure 4:**
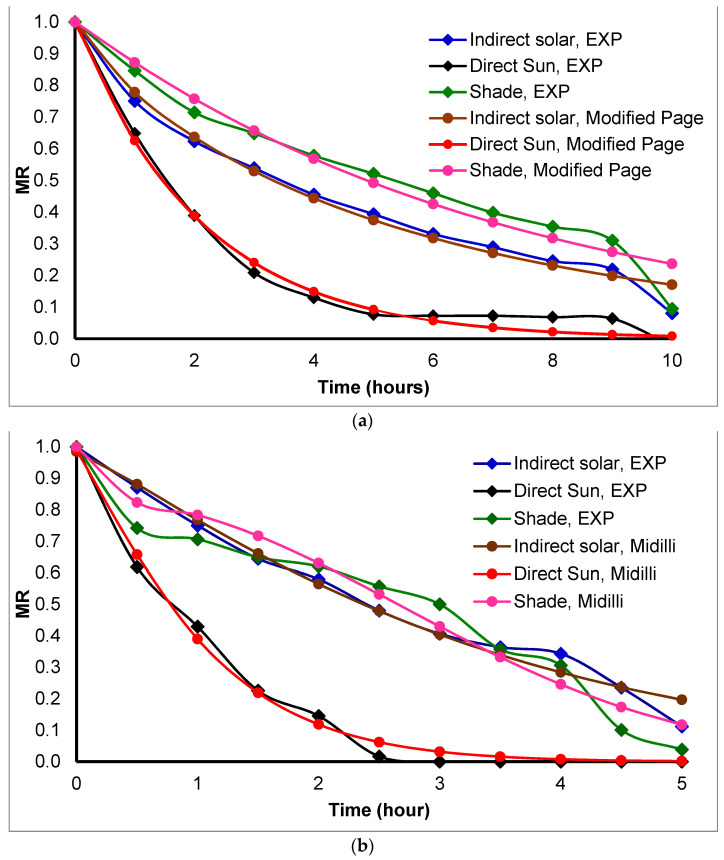
Drying kinetic model resemblance for *Basil* drying during the (**a**) winter and (**b**) summer seasons.

The moisture ratio curves for *Basil* in Figure 4 show that the best-resembled model for *Basil* drying was modified Page in winter and Midilli-Kucuk in summer. As shown in Table 2 the modified Page model has higher R^2^ = 0.98578 and lower X^2^ = 9.68 × 10^−4^, and RMSE = 9.00 × 10^−4^ in winter and Midilli-Kucuk has higher R^2^ = 0.98538 in and lower X^2^ = 1.26 × 10^−3^, and RMSE = 1.13 × 10^−3^ in summer. On the other hand, using the other two methods sun and shade drying, *Basil* resembled with Midilli-Kucuk model. In addition, Figure 4a shows a different slop for the decrease in moisture ratio after 9 h due to the experiment continuing for two days. Furthermore, Figure 4b shows that the summer drying was faster than winter because of the differences in ambient temperature and relative humidity. Summer drying took 5 h and winter took 10–14 h to reach set moisture content.

**Figure 5:**
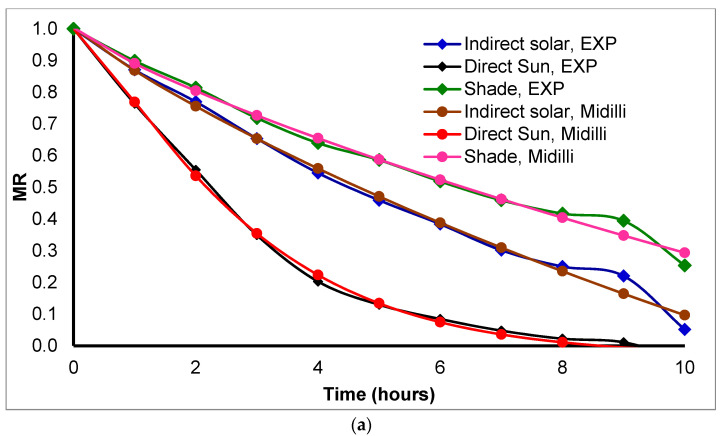

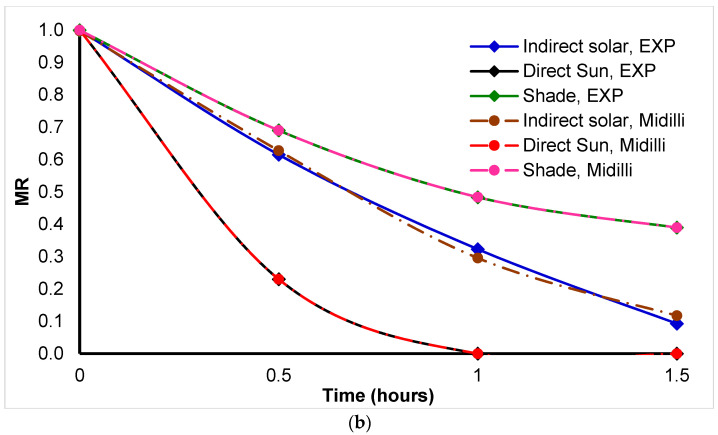
Drying kinetic model resemblance for *Mint* drying during (**a**) winter and (**b**) summer seasons.

The moisture ratio curves presented in Figure 5 show that the best-resembled model for *Mint* herb was Midilli-Kucuk in both seasons (summer, and winter). As shown in Table 2, the best model had higher R^2^ = 0.99536, 0.99248; lower X^2^ = 5.81 × 10^−4^, 5.97 × 10^−4^; and RMSE = 3.79 × 10^−4^, 5.48 × 10^−4^ in the summer and winter seasons, respectively. Furthermore, using the other two drying methods sun and shade drying, *Mint* herbs fitted with Midilli-Kucuk models. In addition, it is clear from Figure 5a that there is a steady decrease in the moisture ratio after 9 h due to the experiment being continued for two days in the wintertime. Figure 5b shows that the summer drying was faster than winter because of the differences in ambient temperature and relative humidity. Summer drying took 1.5 h, and in winter it was 10–14 h to reach the expected moisture content.

The drying process depends on system design, system conditions, and material properties. Controlling all factors at one-time needs a special handling effort. From the previous analyses, the best mathematical model for dried herbs is Midilli-Kucuk based on model resemblance parameters.

### 4.4. Color Change of Medicinal Herbs

**Figure 6:**
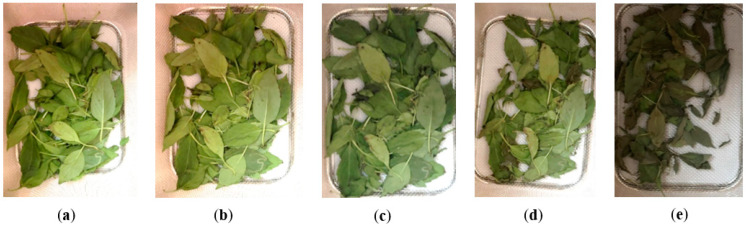
Basil color retention during drying process using indirect solar dryer. (**a**) Fresh, MC= 87.7%. (**b**) 2 h, MC= 47%. (**c**) 4 h, MC= 31.8%. (**d**) 6 h, MC = 21.4%. (**e**) 8 h, MC = 12.6%.

**Figure 7:**
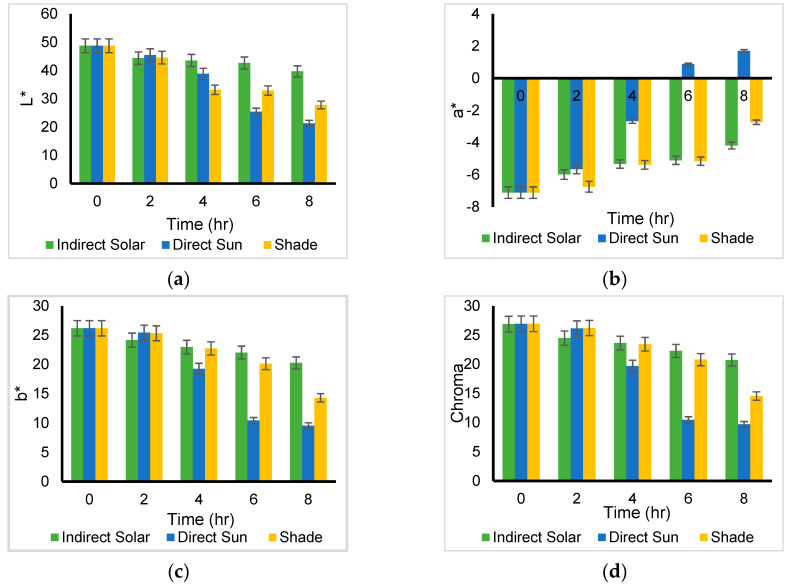

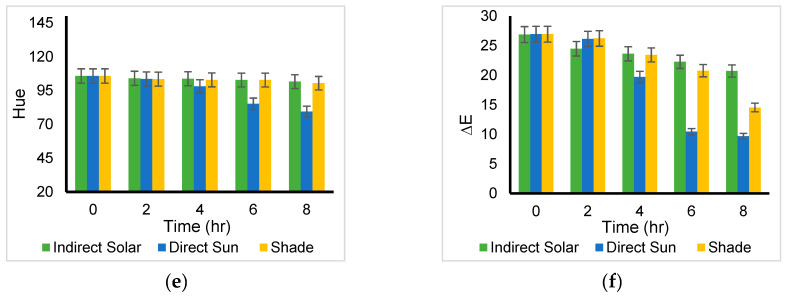
Comparison of *Basil* color parameters by drying method in summer season: ((**a**) L*: Lightness, (**b**) a*: (+) redness/(−) greenness, (**c**) b*: (+) yellowness/(−) blueness, (**d**) Chroma, (**e**) Hue, and (**f**) color difference) degradation during indirect solar-drying (*n* = 3).

The independent variables (time and drying methods) used in the drying process for *basil* herb show a significant difference in the color parameter changes for the greenness (a*) value (*p* < 0.05). Moreover, the independent variable (time) has a significant difference with lightness (L*), yellowness (b*), and chroma values (*p >* 0.05). However, there is no significant difference between the selected independent variables (time and drying methods) and the hue and Dark Green Color Index (DGCI) values. Figure 6 shows that indirect solar drying has lower degradation compared with other methods. The lightness was reduced to 39.67, 21.29, and 27.77 for indirect solar, direct sun, and shade drying, respectively (Figure 7a). Moreover, the drying process influenced the greenish value of all drying methods, but the indirect solar dying has less effect, and the green value reached −4.185 compared with direct sun and shade drying 1.70, and −2.73 (Figure 7b). The green color is represented by negative a* values, while the loss of green coloration is represented by a rise in a* values toward zero. It was assumed that the loss of green color was caused by chlorophyll decomposition during drying because chlorophyll is a green component of herbs. Furthermore, the yellow value was weakened to 20.28 in indirect solar drying, 9.53 in direct sun drying, and 14.29 in shade drying (Figure 7c). In plants, chlorophyll is present as a mixture of blue-green chlorophyll a and yellow-green chlorophyll b [22]. Thus, chlorophyll was considered responsible for the decline in b* values. Furthermore, the hue and Chroma were reduced by 22.9% and 4% in indirect solar drying, respectively (Figure 7d, e). Therefore, the color difference showed a huge change in direct sun drying compared with indirect solar drying due to drying temperature and time (Figure 7f). As discussed, basil leaves color changes could be caused by chlorophyll deterioration. The decomposition of chlorophyll relates to an enzymatic reaction involving chlorophyllase [23]. The current study was matching the result obtained by Yilmaz et al., [24] which indicated that the color reduction was affected by drying temperature, light, and airflow. Furthermore, Yilmaz et al., [24] reported that natural drying has less color loss compared with microwave drying.

**Figure 8:**
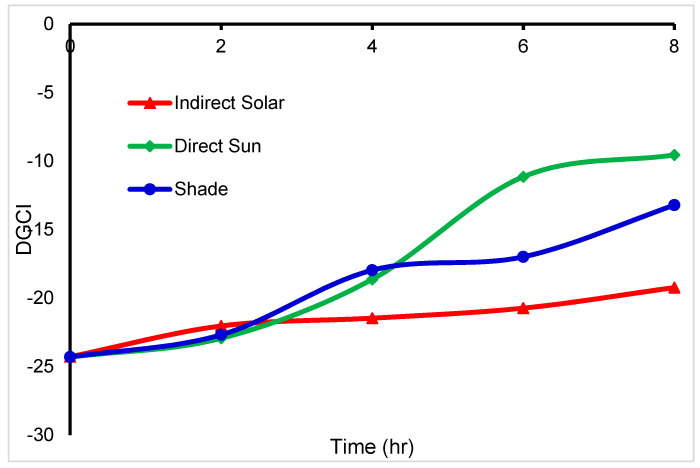
Basil DGCI degradation during different drying methods (*n* = 3).

The DGCI of *basil* was clearly shown in Figure 8, which indicated that was no relationship between DGCI and drying methods used for *basil*.

**Figure 9:**
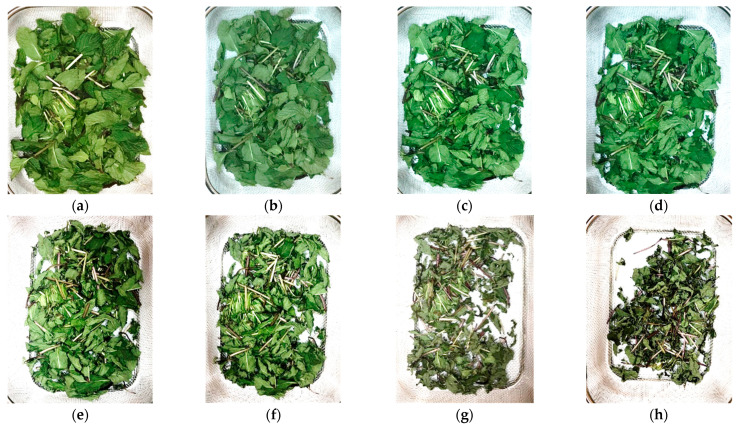
Mint color retention during drying process using indirect solar dryer. (**a**) Fresh, MC = 71%. (**b**) 1 h, MC = 60%. (**c**) 2 h, MC = 52%. (**d**) 3 h, MC = 35%. (**e**) 4 h, MC = 23%. (f) 5 h, MC = 18%. (**g**) 6 h, MC = 15%. (**h**) 6 h, MC =13%.

The color parameter considered is an important factor that indicates the quality of the herbs. In this research, the indirect solar dryer shows good color retention capability in all selected herbs. Figure 9 presents visuals of mint color degradation during the drying process.

**Figure 10:**
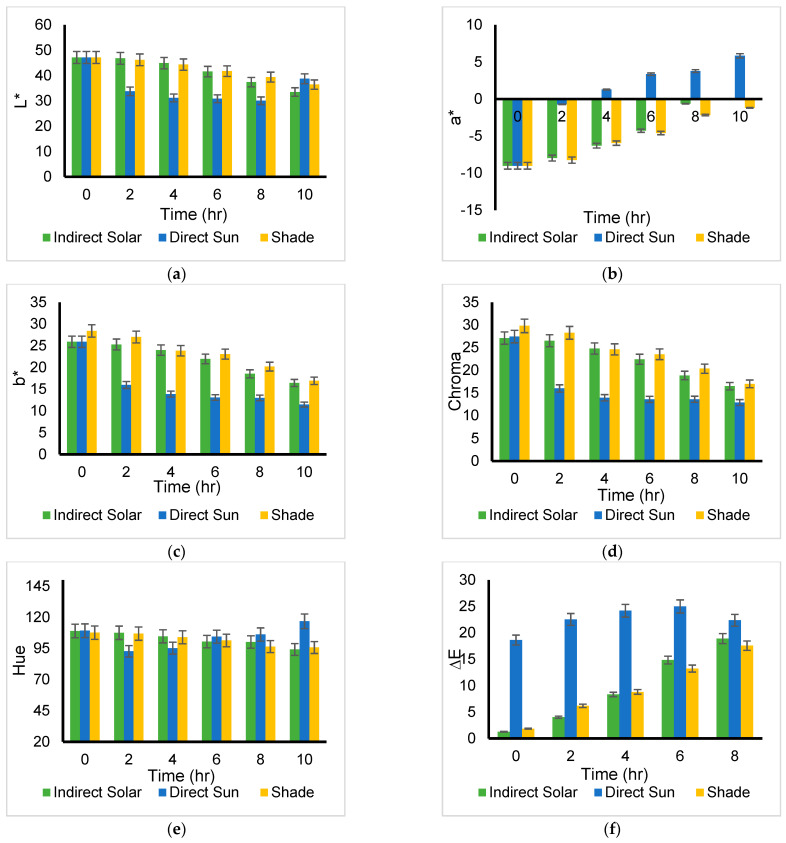
*Mint* color parameters: ((**a**) L*: Lightness, (**b**) a*: (+) redness/(−) greenness, (**c**) b*: (+) yellowness/(−)blueness, (**d**) Chroma, (**e**) Hue, and (**f**) color difference) degradation during indirect solar-drying (*n* = 3).

**Figure 11:**
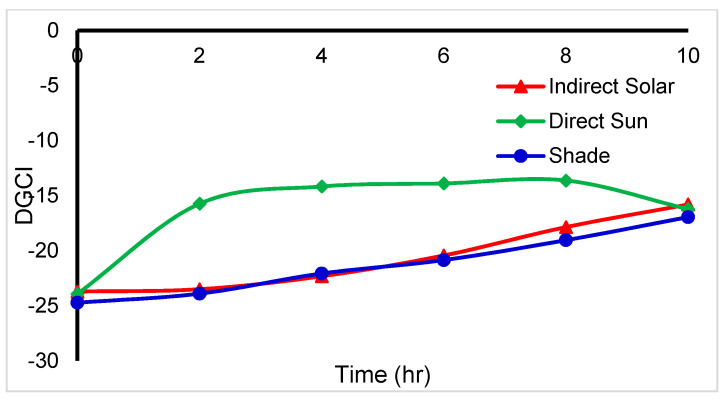
Mint DGCI degradation during different dying methods (*n* = 3).

The independent variables (time and drying methods) used to dry *mint* herb show significant differences in color parameter changes for L*, a*, b*, chroma, and DGCI values (*p* < 0.05). However, no significant difference was observed in the hue values (*p* > 0.05). Figure 10 shows the color data in terms of the L*, a*, and b* values of fresh and dried *mint* leaves. The L* values of the *mint* leaves decreased by drying. The L* values of the sun-dried and shade samples were lower among the indirect solar dying, which was closer to the L* values of the fresh sample (*p* < 0.05). The a* values of the *mint* leave increase during drying from dark green to light green (negative = green), but the direct sun dying sample reaches a high value (positive = red). The b* values of the *mint* leave decrease within the drying process. Direct sun drying shows a lower b* value. Figure 11 shows that indirect solar drying has lower degradation compared with other methods. The lightness was decreased by 29%, 18%, and 22% in indirect solar, direct sun, and shade drying, respectively (Figure 10a). Moreover, the drying process influenced the green value of all drying methods, but the indirect solar dying has less effect, and the green retention reaches 99% compared with direct sun drying (64% (Figure 10b)). Furthermore, the yellow value was weakened by 36% in indirect solar drying, 44% in direct sun drying, and 59% in shade drying (Figure 10c). The hue and Chroma were reduced by 39% and 13% in indirect solar drying, respectively (Figure 10d,e). Therefore, the color difference showed a significant change in direct sun drying compared with indirect solar drying due to drying temperature and time (Figure 10f). Therdthai et al [25] reported that the fresh *mint* had L* = 35.39 ± 1.36, a* = 10.23 ± 0.88, and b* = 26.92 ± 1.01, respectively, which confirmed the current study. Furthermore, Therdthai et al. [25] stated that the best-dried *mint* has a green-yellow color that is reached by increasing L*. High temperatures may cause the magnesium in chlorophyll to be replaced by hydrogen and transform chlorophylls into pheophytins [26]. Although changes in color during drying are inevitable, the changes in color could be reduced by optimizing the drying process parameters, such as drying temperature, time, and air velocity.

The DGCI is a quantitative parameter that shows the greenness level related to the amount of nitrogen in specific leaves. The DGCI of *mint* shown in Figure 11 indicates a sharp increase in direct sun and a smooth increase in indirect solar and shade drying. As a result, the amount of nitrogen is higher in *mint* dried by indirect solar and shade. At the endpoint, the DGCI becomes the same for all drying methods which means it is an unexpected result. Unfortunately, there are no studies found that handled DGCI for dried leaves.

### 4.5. Water Activity of Dried Medicinal Herbs

**Figure 12:**
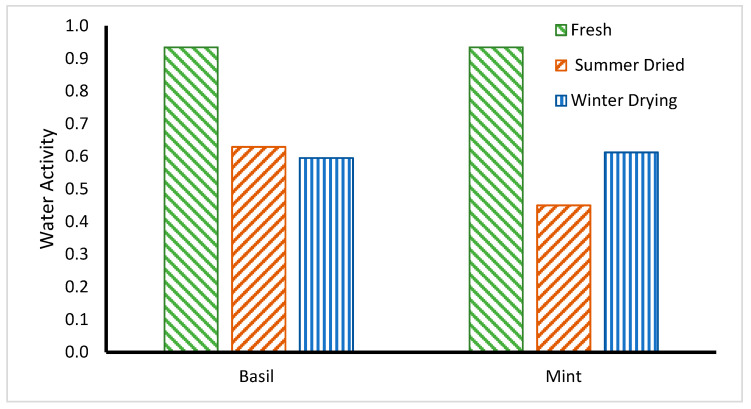
The water activities of *Basil* and *Mint* (*n* = 3).

Water activity is a very reliable indicator used for food preservation and safety by the elimination of microorganism growth, physical and chemical reactions, and spoilage of dry food products. Figure 12 shows a sharp decrease in water activity (A_W_) of dried herbs during the drying process. The A_W_ of the *basil* and *mint* was recorded in winter as 0.595 (initial 0.930) and 0.612 (initial 0.935), respectively. However, the value increased during the summer to 0.629 for *basil*, but the value decreased during summer to 0.44 for *mint*. The water activity of dried *basil* in summer showed a higher value than in the winter due to improper storage conditions, which indicated a possible moisture absorbance. At water activity of more than 0.7, the quality of food products can be affected by moulds, yeasts, bacteria, and other microorganisms [27]. Food quality and stability can be maintained through drying, which lowers water activity by lowering moisture content and prevents spoiling and contamination during storage.

### 4.6. Total Phenol Content of Medicinal Herbs

**Figure 13:**
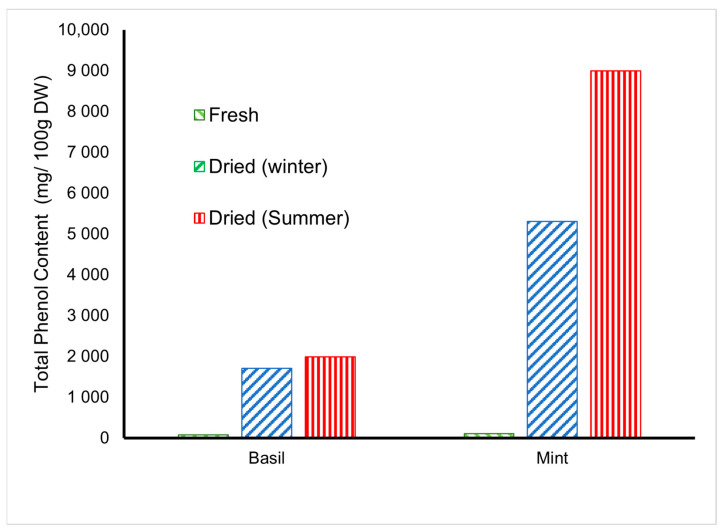
The Total Phenol Content changes in medicinal dried herbs (*n* = 3).

Figure 13 shows the total phenols contents found for *basil* and *mint* leaves dried in an indirect solar dryer. It is observed that the increase in total phenol content compares with the increase in temperature. The value of phenol content increased from 81 to 1705 and 110 to 8994 mg Caffeic acid/100 g dry matter for *basil* and *mint*, respectively. According to the figure, the lowest total phenolic content was found in the fresh product. With drying, as the water in the products evaporates, the product’s concentration increases, that is, a dry product that weighs the same as the fresh product has about six to seven times more concentrated than fresh. Jimenez-Garcia et al. [28] investigated thyme’s phenolic content, dried by convective and microwave drying methods, compared with fresh produce. The results were in agreement with this study. These results are consistent with the Turgay et al. [29] results, which indicated the increment in *basil*’s total phenolic content. Xylia et al., [30] also found that the total phenol content of rosemary was increased using different heat treatments. Rababah et al. [31] stated that the drying process tends to decrease 30% of polyphenols located in the total phenol content. Furthermore, there is an inverse relation between color lightering and total phenol content; as L* increases, the decrease in total phenol content [32]. The moderate heat treatment (≤50 °C) used by the solar dryer may have determined the cleavage of the phenolic sugar glycosidic bonds and the formation of phenolic aglycons, thus leading to higher values of the total phenolic content [33].

### 4.7. Antioxidant Capacity of Medicinal Herbs

**Figure 14:**
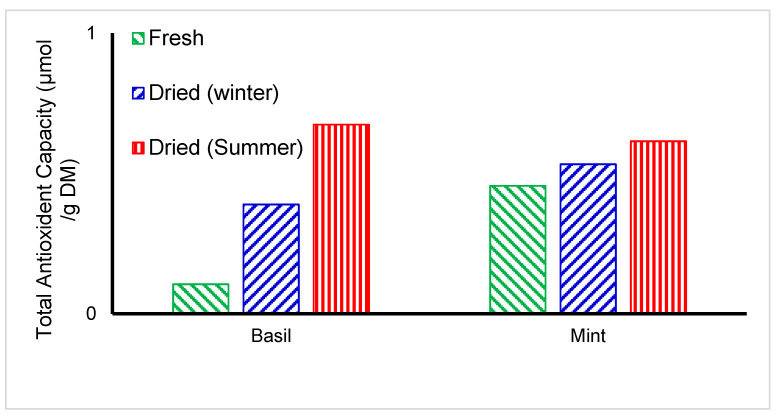
Total Antioxidant Capacity of medicinal dried herbs after and before drying (*n* = 3).

The antioxidant capacity of fresh and indirect solar-dried *basil* and *mint* samples is shown in Figure 14. Both seasons show an increase in the antioxidant capacity of dried medicinal herbs. The antioxidant capacity reached 0.67 (initial 0.105) and 0.61 (initial 0.455) μmol/g dry mater for *basil* and *mint*, respectively. Dried herbs had better antioxidant activity than fresh herbs. Enzymes were inactivated due to decreased water activity and thus retained high antioxidant capacity in the dried samples. The reducing property and free radical ability of both fresh and dried green leafy vegetables were determined by Oboh et al. [18]. The ability of the vegetables to reduce Fe (III) to Fe (II) was used to determine their reducing property.

## 5. Conclusions

The developed stand-alone indirect solar dryer functioned well and was found to be the first such attempt for medicinal herbs drying in Oman using the black body radiation concept. The color parameters changed negatively with drying time in all drying methods, but indirect solar drying showed less change compared to direct sun and shade drying. The main focus was on a*, Chroma, and dark green color index values, which shows the retention of the herb’s green color and, thus, the physicochemical properties. A higher reduction of water activity occurred for medicinal herbs in the winter at 0.46 and 0.57 for *basil* and *mint*, respectively, while the higher retention of water activity for *mint* occurred during the summer season (0.44). The best water activity to prevent microorganism growth is reported to be less than 0.6. The numerical value of antioxidant capacity for dried herbs was found 0.61–0.67 µmol/g dry mater. When considering the possible contamination of dust and insects under sun or shade drying, this low-cost stand-alone renewable energy used drying concept can be disseminated for implementation in small and medium enterprises in Oman. The future direction of this research is studying the factors affecting the drying process to reduce the drying time and improve physiochemical quality and energy efficiency.

## Figures and Tables

**Table 1:** Mathematical models used to describe the drying kinetics of leafy vegetables.

Model Name	Model Equation	References
Lewis	MR=exp(−Kt)	[11]
Modified Page (I)	$MR=\exp\left|-{\left(Kt\right)}^{n}\right|$	[12]
Midilli and Kucuk	$MR=a\;exp\left(-Kt^{n}\right)+bt$	[13]

**Table 2:** Statistical analysis and model parameters of resembled drying kinetic models for medicinal herbs.

			Midilli and Kucuk			Lewis			Modified Page		
			R^2^	X^2^	RMSE	R^2^	X^2^	RMSE	R^2^	X^2^	RMSE
Basil	Summer	Indirect	0.98167	1.26 × 10^−3^	1.13 × 10^−3^	0.98122	1.46 × 10^−3^	1.46 × 10^−3^	0.98538	1.29 × 10^−3^	1.15 × 10^−3^
		Direct	0.97956	2.08 × 10^−3^	1.84 × 10^−3^	0.97045	2.61 × 10^−3^	2.61 × 10^−3^	0.99302	2.09 × 10^−3^	1.14 × 10^−3^
		Shade	0.90394	7.39 × 10^−3^	5.97 × 10^−3^	0.88969	8.53 × 10^−3^	8.53 × 10^−3^	0.89823	8.93 × 10^−3^	7.96 × 10^−3^
	Winter	Indirect	0.96632	2.26 × 10^−3^	1.99 × 10^−3^	0.97758	1.59 × 10^−3^	1.59 × 10^−3^	0.98578	9.68 × 10^−4^	9.00 × 10^−4^
		Direct	0.99227	7.04 × 10^−4^	6.33 × 10^−4^	0.98972	8.70 × 10^−4^	8.70 × 10^−4^	0.98976	8.70 × 10^−4^	7.97 × 10^−4^
		Shade	0.96093	2.27 × 10^−3^	2.23 × 10^−3^	0.96564	2.59 × 10^−3^	2.59 × 10^−3^	0.96586	2.59 × 10^−3^	2.37 × 10^−3^
Mint	Summer	Indirect	0.99536	5.81 × 10^−4^	3.79 × 10^−4^	0.96953	2.48 × 10^−3^	2.48 × 10^−3^	0.99533	5.83 × 10^−4^	3.81 × 10^−4^
		Direct	0.96712	7.75 × 10^−3^	4.28 × 10^−3^	0.95222	6.28 × 10^−3^	6.28 × 10^−3^	0.96712	7.17 × 10^−1^	4.28 × 10^−3^
		Shade	1.00000	3.91 × 10^−13^	2.78 × 10^−13^	0.98853	4.56 × 10^−4^	4.56 × 10^−4^	0.99666	1.70 × 10^−4^	1.34 × 10^−4^
	Winter	Indirect	0.99248	5.97 × 10^−4^	5.48 × 10^−4^	0.97041	2.42 × 10^−3^	2.42 × 10^−3^	0.98719	1.22 × 10^−3^	1.08 × 10^−3^
		Direct	0.99781	2.42 × 10^−4^	2.15 × 10^−4^	0.97973	1.87 × 10^−3^	1.87 × 10^−3^	0.99674	3.58 × 10^−4^	3.15 × 10^−4^
		Shade	0.99221	4.36 × 10^−4^	4.04 × 10^−4^	0.99217	4.36 × 10^−4^	6.15 × 10^−4^	0.99336	5.83 × 10^−4^	5.26 × 10^−4^

R^2^: Coefficient of determination, X^2^: chi-square, and RMSE: Root Mean Square Error.

## Data Availability

Data is contained within the article.
